# Prevalence and characterisation of non-cholerae *Vibrio* spp. in final effluents of wastewater treatment facilities in two districts of the Eastern Cape Province of South Africa: implications for public health

**DOI:** 10.1007/s11356-014-3461-z

**Published:** 2014-08-29

**Authors:** Anthony I. Okoh, Timothy Sibanda, Vuyokazi Nongogo, Martins Adefisoye, Osuolale O. Olayemi, Nolonwabo Nontongana

**Affiliations:** Applied and Environmental Microbiology Research Group (AEMREG), Department of Biochemistry and Microbiology, University of Fort Hare, P Bag X1314, Alice, Eastern Cape 5700 South Africa

**Keywords:** *Vibrio*, Public health, Wastewater, Antibiogram, Prevalence

## Abstract

Vibrios and other enteric pathogens can be found in wastewater effluents of a healthy population. We assessed the prevalence of three non-cholerae vibrios in wastewater effluents of 14 wastewater treatment plants (WWTP) in Chris Hani and Amathole district municipalities in the Eastern Cape Province of South Africa for a period of 12 months. With the exception of WWTP10 where presumptive vibrios were not detected in summer and spring, presumptive vibrios were detected in all seasons in other WWTP effluents. When a sample of 1,000 presumptive *Vibrio* isolates taken from across all sampling sites were subjected to molecular confirmation for *Vibrio*, 668 were confirmed to belong to the genus *Vibrio*, giving a prevalence rate of 66.8 %. Further, molecular characterisation of 300 confirmed *Vibrio* isolates revealed that 11.6 % (35) were *Vibrio parahaemolyticus*, 28.6 % (86) were *Vibrio fluvialis* and 28 % (84) were *Vibrio vulnificus* while 31.8 % (95) belonged to other *Vibrio* spp. not assayed for in this study. Antibiogram profiling of the three *Vibrio* species showed that *V. parahaemolyticus* was ≥50 % susceptible to 8 of the test antibiotics and ≥50 % resistant to only 5 of the 13 test antibiotics, while *V. vulnificus* showed a susceptibility profile of ≥50 % to 7 of the test antibiotics and a resistance profile of ≥50 % to 6 of the 13 test antibiotics. *V. fluvialis* showed ≥50 % resistance to 8 of the 13 antibiotics used while showing ≥50 % susceptibility to only 4 antibiotics used. All three *Vibrio* species were susceptible to gentamycin, cefuroxime, meropenem and imipenem. Multiple antibiotic resistance patterns were also evident especially against such antibiotics as tetracyclin, polymixin B, penicillin G, sulfamethazole and erythromycin against which all *Vibrio* species were resistant. These results indicate a significant threat to public health, more so in the Eastern Cape Province of South Africa which is characterised by widespread poverty, with more than a third of the population directly relying on surface water sources for drinking and daily use.

## Introduction

Municipal wastewater, even if treated, may still contain a wide range of pathogens that are excreted by diseased humans (Arceivala 1997; Wen et al. 2009), contributing to increased densities of pathogens in the receiving water bodies. Surface water bodies are major sources of potable water whose unabated contamination has led to many water-related disease outbreaks in the past (Mishra et al. 2004; Nair et al. 2004; Obi et al. 2004). Although wastewater treatment technologies can, with optimised performance, reduce bacterial and viral pathogens by approximately 90 % (Asano and Levine 1998; Jiménez et al. 2004), it is not possible for the microbial quality of the effluents to match the microbial quality of the water in the receiving water bodies. Discharge of effluents will, therefore, despite the level of treatment, potentially alter the microbial content of the receiving water bodies (Drury et al. 2013). Previous studies show that some *Vibrio* species survive the activated sludge-based wastewater treatment process as free cell and as plankton-associated entities (Igbinosa et al. 2009, 2011), suggesting that the provision of wastewater treatment facilities does not, in itself, ensure satisfactory effluent water quality. The most common clinical presentation of *Vibrio* infection is self-limited gastroenteritis, but wound infections and primary septicaemia may also occur (Levine and Griffin 1993). Healthy carriers of *Vibrio cholerae* excrete vibrios intermittently, with chronic convalescent carriers shedding vibrios intermittently for periods of 4 to 15 months (Nevondo and Cloete 2001). Survival of vibrios in the aquatic environment relates sharply to various chemical, biological and physical characteristics of the aquatic milieu, with *V. cholerae* known to remain viable in surface waters for periods ranging from 1 h to 13 days, while faecal contamination from victims of epidemics and healthy carriers may continue to reinforce their concentrations in water (Nevondo and Cloete 2001). As a result, cholera and cholera-like infections continue to be a substantial health burden in developing countries, especially in Africa and Asia, compromising the primary health of vulnerable members of society (Bourne and Coetzee 1996; Pegram et al. 1998; Mackintosh and Colvin 2003). *Vibrio* species have been incriminated in cases of diarrhoea, accounting for a substantial degree of morbidity and mortality in different age groups worldwide (Obi et al. 2004). The most notable of *Vibrio* pathogens are *V. cholerae*, *Vibrio parahaemolyticus*, *Vibrio vulnificus* and *Vibrio fluvialis* (CDC Centers for Disease Control and Prevention 1999; Finkelstein et al. 2002; Kothary et al. 2003; Chakraborty et al. 2006) which are mainly transmitted via water and food. They all cause diarrhoea, but in entirely different ways; *V. vulnificus* and *V. parahaemolyticus* are invasive organisms, affecting primarily the colon, while *V. cholerae* is non-invasive, affecting the small intestine through secretion of an enterotoxin (Todar 2005), and is the etiologic agent of cholera. The clinical symptoms of *V. fluvialis* gastroenteritis are similar to cholera with the additional manifestation of bloody stools which is suggestive of an invasive pathogen (Oliver and Kaper 2001). Other vibrios like *Vibrio alginolyticus*, *Vibrio cincinnatiensis*, *Vibrio furnisii*, *Vibrio harveyi*, *Vibrio metschnikovii* and *Vibrio mimicus* have occasionally been reported as causes of human infections (Farmer and Hickman-Brenner 1992; Abbott and Janda 1994; Carnahan et al. 1994). However, of all the *Vibrio* species which have been associated with illness in humans, the most important are *V. cholerae* subgroups O1 and O139, the causative agents of epidemic cholera (Heymann 2008). Heidelberg et al. (2002) have reported large numbers of vibrios, about 4.3 × 10^6^/mm^2^, attached to the external surface of plankton (zooplankton and phytoplankton), pointing to a close association between vibrios and planktons. This association has also been observed in municipal wastewaters (Ahmadi et al. 2005; Chindah et al. 2007; Mukhopadhyay et al. 2007). *V. fluvialis*, in particular, has been identified as an important cause of cholera-like bloody diarrhoea and primary septicaemia in immunocompromised individuals, especially in underdeveloped countries with poor sanitation (Igbinosa and Okoh 2010). This organism has been isolated from treated wastewater effluents in South Africa (Igbinosa et al. 2009), and there are reports linking it to food poisoning (Kobayashi and Ohnaka 1989), especially due to consumption of raw shellfish (Levine and Griffin 1993). While adequate and timely rehydration therapy remains the gold-standard treatment for cholera and cholera-like bloody diarrhoea (Heymann 2008), antimicrobials are also prescribed for the management of severe cases in order to shorten the duration of illness and reduce the volume of rehydration solution required. However, some *Vibrio* strains are resistant to a number of antimicrobials including tetracycline, co-trimoxazole, trimethoprim and sulfamethoxazole. This resistance to antimicrobials, in addition to other properties such as virulence factors and ability to cause epidemics, makes vibrios pathogens of public health concern. Knowledge of the prevalence and antimicrobial resistance profile of local strains is, therefore, important for the management of complicated cases in the case of an epidemic. At times, a cholera outbreak is reported without any clear linkage of the index case to neighbouring countries or travel to affected areas. This usually leaves health authorities asking themselves where the cholera causing bacteria could have come from. We hypothesise that such outbreaks could be related to either persistence of organisms in free-living, altered or adapted forms capable of reverting to a pathogenic variety or to continuous year-round transmission by sub-clinical cases or a combination of both. Routine analysis of the microbial quality of treated wastewater effluents is therefore warranted in order to maintain the microbial load of receiving water bodies within acceptable limits for both human use and lotic ecosystems survival. There is, however, paucity of information on the molecular epidemiology of vibrios in the aquatic milieu of the Eastern Cape Province (ECP) of South Africa. Compounding the challenge is the production of poor-quality effluents by wastewater treatment plants in the ECP which is acknowledged as mostly non-urban, poor and without adequate infrastructure (Mohale 2003; BLACKSASH 2010). Also, the documentation of final effluent compliance of the wastewater treatment plants to set guidelines with respect to bacteriological quality remains poor in the province. This study was, therefore, aimed at assessing the prevalence of non-cholerae vibrios in the final effluents of 14 wastewater treatment facilities in the ECP of South Africa, characterising them into species and determining their antibiogram properties and evaluating the public health implications of the findings. While there are at least 12 pathogenic *Vibrio* species recognised to cause human illness (Janda et al. 1988), this work was based on the prevalence and characterisation of *V. vulnificus, V. fluvialis* and *V. parahaemolyticus.*

### Methodology

#### Description of study area

**Fig. 1:**
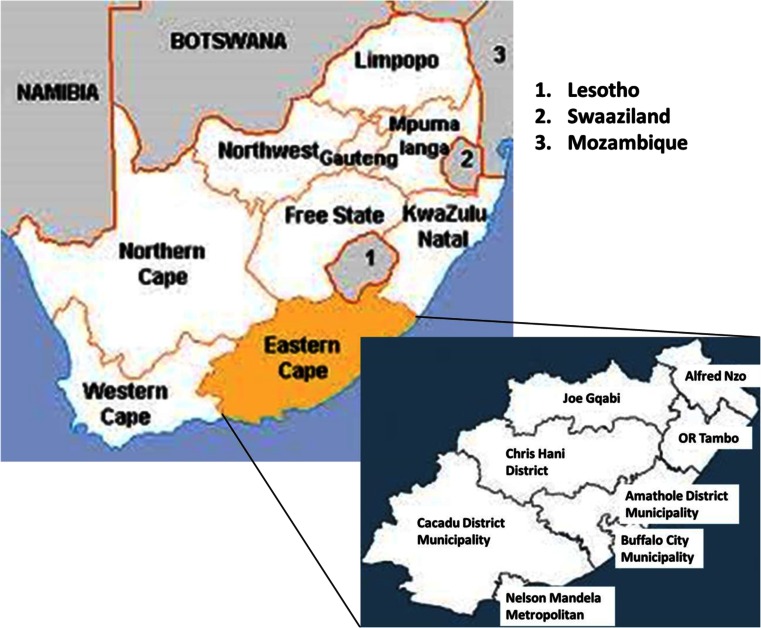
A map showing the seven district municipalities of the ECP. Sampling sites were selected in Chris Hani and Amathole district municipalities (http://www.worldlicenseplates.com/world/AF_ZAEC.html)

Fourteen (14) wastewater treatment plants (WWTP) were selected in Amathole and Chris Hani district municipalities of the ECP in South Africa (Fig. 1). The choice of WWTP was influenced by the need to ensure that plants were not located more than 3-h drive from the University of Fort Hare in Alice, such that samples could be taken to the laboratory for analysis within 6 h of collection. The ECP borders the provinces of the Western Cape, Northern Cape, Free State and KwaZulu-Natal, as well as Lesotho in the north. The province is mostly rural with a high percentage of people living in poverty (67.4 %) and a very low Human Development Index (HDI) of 0.52 (BLACKSASH 2010; ECSECC 2011). It is the second largest province in South Africa and mainly comprised of rural settlements with little or no adequate sanitary facilities, with about 36 % of the population directly reliant on surface water sources for domestic use (ECSECC 2011). The ECP is divided into seven district municipalities, namely, Alfred Nzo, Amathole, Chris Hani, Joe Gqabi, O.R. Tambo, Cacadu and the Nelson Mandela Metropolitan Municipality. The Amathole District Municipality includes the Buffalo City Municipality. Due to the confidential nature of this work, the sampling sites were designated as WWTP1 to WWTP14, and geographical coordinates could equally not be given. All selected WWTPs discharge their treated effluents directly into rivers. Samples were collected with permission from the Amathole and Chris Hani district municipalities.

#### Sample collection and isolation of presumptive *Vibrio* organisms

Wastewater final effluent samples were collected once a month for a period of 12 months starting in August 2012 to July 2013. Samples were collected in sterile 2-l polypropylene bottles containing 0.1 % of a 3 % (*w*/*v*) solution of sodium thiosulphate for sample dechlorination and taken to the Applied and Environmental Microbiology Research Group (AEMREG) laboratory at the University of Fort Hare in Alice, South Africa, in cooler boxes containing ice, for analysis within 6 h of collection. Samples were serially diluted and concentrated on nitrocellulose membrane filters (0.45-μm pore size, Millipore) by passing 100 ml of each dilution through the filter using the membrane filtration technique as recommended by Standard (2005). The filters were then placed onto agar plates containing thiosulphate citrate bile salts sucrose agar (TCBS agar). For the purposes of quality control, the spread plate technique was also employed where known (100 μl) volumes of effluent samples were spread on TCBS agar as previously described by Igbinosa et al. (2011). Green and yellow colonies were identified and enumerated as presumptive *Vibrio* isolates. Counts were converted to Log_10_ values and clustered into seasons where spring composed of August 2012–October 2012, summer (November 2012–January 2013), autumn (February 2013, March 2013 and April 2013) and winter (May 2013–July 2013). Presumptive *Vibrio* colonies were then isolated, purified and subjected to Gram staining and oxidase test. Gram-negative, oxidase positive isolates were selected and preserved in 20 % glycerol at −80 °C until further analysis.

#### Molecular confirmation of *Vibrio* species

To extract DNA, single colonies of 18–24 h old presumptive *Vibrio* cultures grown on nutrient agar plates at 37 °C were picked, suspended in 200 μl of sterile distilled water and the cells lysed using an AccuBlock (Digital dry bath, Labnet) for 15 min at 100 °C as described by Maugeri et al. (2006). The cell debris was removed by centrifugation at 11,000×*g* for 2 min using a MiniSpin microcentrifuge (Lasec, RSA). Five microlitre (5 μl) aliquots of the cell lysates were used as template DNA in polymerase chain reaction (PCR) assays immediately after extraction to determine the molecular identity of the isolates. To confirm if the isolates belonged to the genus *Vibrio*, the primer set V16S-700F (CGG TGA AAT GCG TAG AGA T) and V16S-1325R (TTA CTA GCG ATT CCG AGT TC) targeting the 16S ribosomal RNA (rRNA) gene (663 bp) was used in PCR assays as described by Kwok et al. (2002).

*Vibrio* species identification was done using species-specific primers targeting specific sequences within the 16S rRNA as described by Kim et al. (1999). For *V. parahaemolyticus*, the primer set Vp. flaE-79F (GCA GCT GAT CAA AAC GTT GAG T) and Vp. flaE-934R (ATT ATC GAT CGT GCC ACT CAC) targeting the 897 bp *flaE* gene was used as described by Tarr et al. (2007). *V. vulnificus* was identified using the primer set Vv. hsp-326F (GTC TTA AAG CGG TTG CTG C) and Vv. hsp-697R (CGC TTC AAG TGC TGG TAG AAG) targeting the *hsp60* gene (410 bp) as described by Wong and Chow (2002), while the primer set Vf toxR-F (GAC CAG GGC TTT GAG GTG GAC GAC) and Vf toxR-R (AGG ATA CGG CAC TTG AGT AAG ACTC) was used to identify *V. fluvialis* targeting the 217 bp *toxR* gene as previously described (Osorio and Klose 2000; Chakraborty et al. 2006). In all, 300 confirmed *Vibrio* isolates were randomly selected from a pool of 668 confirmed *Vibrio* isolates taken from across all sampling sites and characterised into these three pathotypes using PCR. *V. parahaemolyticus* DSM 11058, *V. fluvialis* DSM 19283 and *V. vulnificus* DSM 11507 were used as positive control strains.

#### Antibiogram characterisation

All *Vibrio* isolates that were positively identified to belong to any of the three pathotypes were subjected to antimicrobial susceptibility testing using the following antibiotics: imipenem, nalidixic acid, erythromycin, sulfamethoxazole, cefuroxime, penicillin G, chloramphenicol, polymixin B, trimethoprim-sulfamethoxazole, tetracycline, gentamicin, meropenem and trimethoprim. Ciprofloxacin and doxycycline have been the antibiotics of choice for adults (except pregnant women) (Steinberg et al. 2001), while erythromycin and trimethoprim-sulfamethoxazole have been recommended for children and pregnant women. These antimicrobial agents are, however, no longer recommended as first-line therapy because of increasing global antimicrobial resistance (Gilbert et al. 1999), and whenever possible, treatment protocols should be based on local antibiogram data (Centers for Disease Control and Prevention 2013).

#### Statistical analysis

An independent samples *t* test (IBM SPSS version 20) was used to compare the mean presumptive *Vibrio* counts of all the WWTPs and also the mean presumptive *Vibrio* counts obtained in each of the seasons. Differences were deemed significant at *P* < 0.05.

## Results and discussion

**Fig. 2:**
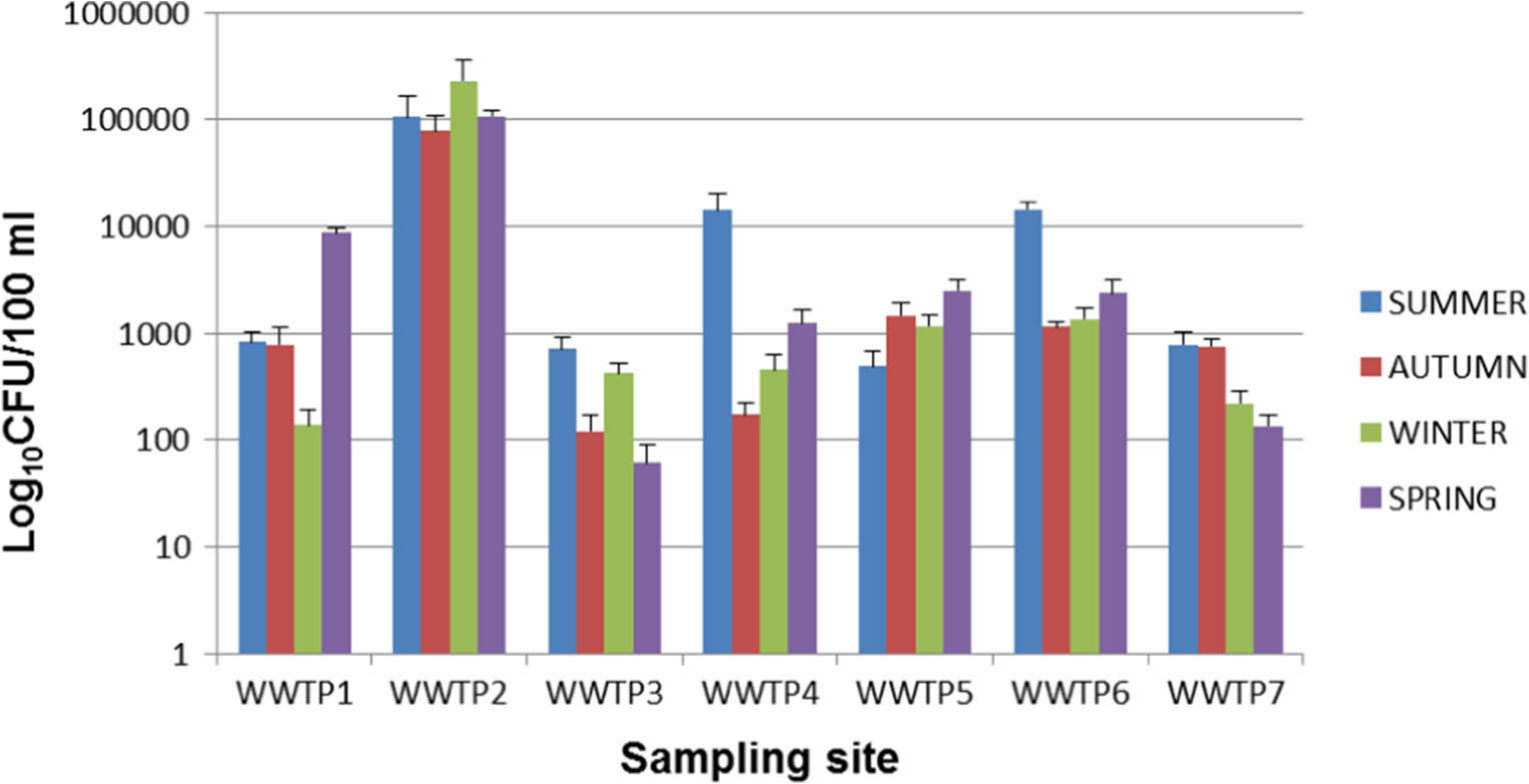
Seasonal presumptive *Vibrio* counts for WWTP1-7

**Fig. 3:**
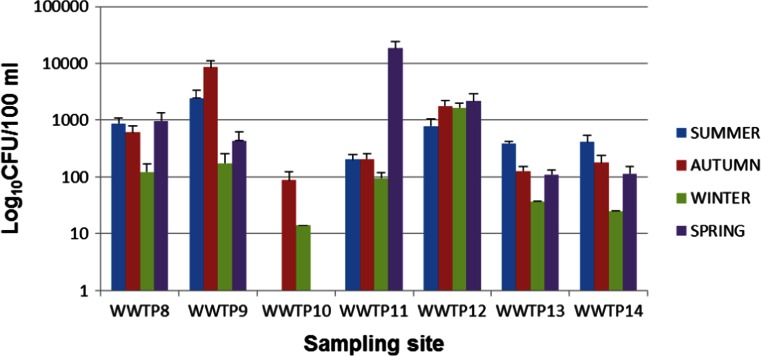
Seasonal presumptive Vibrio counts for WWTP8-14

Presumptive *Vibrio* organisms were isolated in all seasons and at all sampling sites with the exception of WWTP10 where presumptive *Vibrio* was not detected in summer and spring. Gastrointestinal pathogenic microorganisms do not occur as a natural part of the normal intestinal microbiota (Gerritsen et al. 2011). Their presence in wastewater, therefore, could be dependent on the number of infected people in the population contributing to the wastewater flow. The presumptive *Vibrio* counts for all sampling sites were expressed in Log_10_ values and are presented in Figs. 2 and 3. The error bars on these figures represent the standard deviations since each of the readings is an average of the counts of 3 months constituting each season. Significantly higher presumptive *Vibrio* counts were obtained in samples from WWTP2 (*P* < 0.05), while samples from WWTP10 had significantly lower counts compared to the rest of the WWTPs. While other studies have reported a reduction in environmental *Vibrio* densities during winter as compared to other seasons (DePola et al. 2003; de Souza Costa Sobrinho et al. 2010), the trend was different in our case as statistical analysis showed that there was no significant difference in *Vibrio* densities obtained in different seasons.

South Africa has been plagued by outbreaks of *Vibrio-*related waterborne infections that are suspected to be linked to inefficiently treated effluents discharge from wastewater treatment facilities (Igbinosa et al. 2011).

When a randomised sample of 1,000 presumptive *Vibrio* isolates were subjected to PCR confirmation for *Vibrio* organisms, 668 isolates tested positive, giving an overall *Vibrio* prevalence rate of 66.8 %. As at the time when this study was carried out, there was no known outbreak of cholera or cholera-like diarrhoea in the ECP and, specifically, in the two districts serviced by the selected 14 WWTPs and yet *Vibrio* was isolated all the same. *Vibrio* species are not normal biota for human beings, as some *E. coli* are, neither are they normal biota for fresh water environments, and positive results from this work strongly point to either unreported sporadic incidents of infection within the communities or the existence of healthy *Vibrio* carriers intermittently shedding vibrios into the environment as suggested by Nevondo and Cloete (2001). Similar findings were obtained by Jackson S. Beney C (2000) who managed to detect potentially virulent *V. cholerae* in freshwater environments despite the absence of clinical cases in the host population for some time. Harris et al. (2012) also stated that some patients can even be infected with *V. cholerae* O1 or O139 and yet show no symptoms but then tend to shed the organism into the environment, even for only a few days, explaining why vibrios can be isolated in wastewater effluents in a non-*Vibrio* and/or non-cholera epidemic area. Once these vibrios get into environmental water, they convert to conditionally viable environmental cells within 24 h (Faruque et al. 2005; Nelson et al. 2008). Such vibrios are infectious on reintroduction into a human body, but the infectious dose in this form is not known (Harris et al. 2012). This becomes a major public health time bomb in underdeveloped areas like the ECP where, as of 2011, about 36 % of the population still got their drinking water directly from rivers and streams (ECSECC 2011). An excerpt from Water Supply, Sanitation and Hygiene (WASH 2010) summed up the challenge as follows,

“*Many of South Africa’s municipal wastewater treatment plants (WWTP) are not performing to acceptable water quality standards …. A lack of good-quality drinking water leads to health problems, which is serious, given the fact that many poor citizens source water directly from the rivers, where not only municipalities …. Since South Africa does not have large rivers, the discharged effluents concentrate into small watercourses*”

If, on the average, every litre of effluent contains about 1,000 presumptive *Vibrio* organisms, as was the case in this study, and the lowest effluent volume produced by a treatment plant is 0.63 ml/day (WWTP7), there will be a daily addition of about 6.3 × 10^8^ presumptive *Vibrio* cells into the environment. The resultant health risk to those who drink untreated water directly from the receiving watercourses can be exacerbated if the receiving watercourses are small as this will minimise the dilution capacity and result in concentration of potentially virulent organisms, increasing the risk of illness in the case of raw water ingestion. While there is no available data to show the proportion of effluent in the receiving rivers’ annual flow volumes in the ECP or in South Africa in general, the annual flow of the Chicago Area Waterway System (which includes all segments of the Chicago River as well as the North Shore Channel) comprises more than 70 % of treated municipal wastewater effluent (Illinois Department of Natural Resources 2011). While the WWTPs in the ECP may not be as numerous and big as to contribute to as high a percentage of the river flow volume, chances are that they may not be as efficient in pathogen removal, taking into account the excerpt from WASH (2010). Other factors that may increase or upset the risk include the infectious dose of the organisms, the presence or absence of virulent factors in the said vibrios and whether or not people filter their water at home before they drink it. Besides direct consumption of untreated surface water, vibrios concentrate in the gut of filter-feeders such as oysters, clams and mussels, where they multiply (Iwamoto et al. 2010). While thorough cooking will destroy these organisms, oysters are often eaten raw and are the most common food associated with *V. parahaemolyticus* infection in the USA (Hlady 1997). Our findings, though not complemented by assessments for virulence and antibiotic resistance genes, indicate that the potential for disease in the community and call for pro-active rather than reactive measures if public health is to be preserved and unnecessary loss of life avoided.

**Fig. 4:**
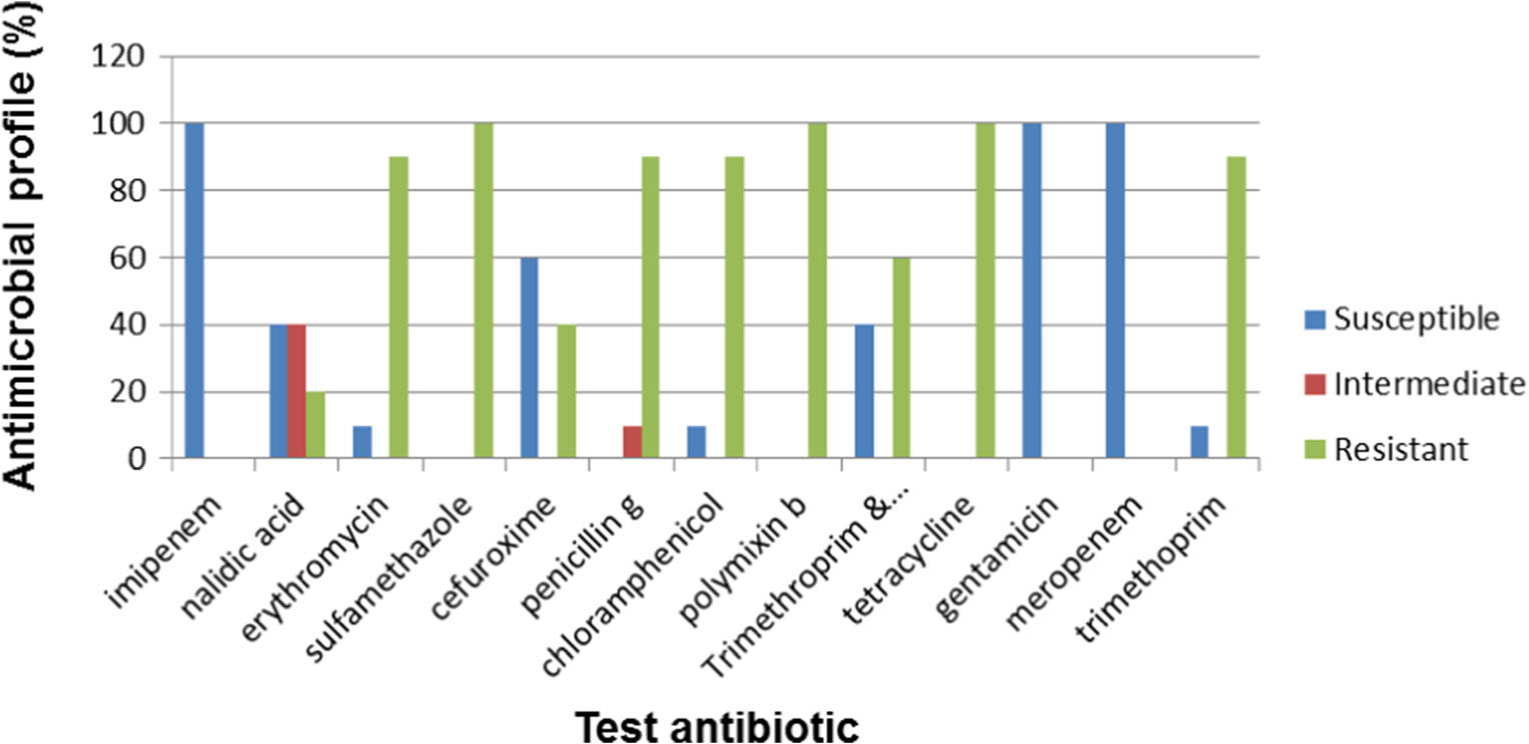
Antimicrobial profile for *V. fluvialis* (*n* = 35)

When 300 of the 668 confirmed *Vibrio* isolates were further screened into species, 11.6 % (35) were confirmed to be *V. parahaemolyticus*, 28.6 % (86) were confirmed to be *V. fluvialis* and 28 % (84) were confirmed to be *V. vulnificus*, while 31.8 % (95) belonged to other *Vibrio* spp. not assayed for in this study. When these confirmed *Vibrio* pathotypes were subjected to antibiogram profiling, *V. fluvialis* showed ≥50 % resistance to 8 of the 13 antibiotics used while showing ≥50 % susceptibility to only 4 antibiotics used (Fig. 4).

Similar results have been reported by Okoh and Igbinosa (2010) whose work showed 100, 92, 90, 70 and 80 % resistances by *V. fluvialis* isolates from rural-based WWTPs to trimethoprim, cephalothin, penicillin, cotrimoxazole and streptomycin, respectively, positioning it as an emerging pathogen in the ECP of South Africa. Antimicrobial resistance has become a major medical and public health problem as it has direct links with disease management and containment. This is reflected by the increase in the fatality rate from 1 to 5.3 % after the emergence of drug resistance strains in Guinea-Bissau during the cholera epidemic of 1996–1997 (Dalsgaard et al. 2000).

**Fig. 5:**
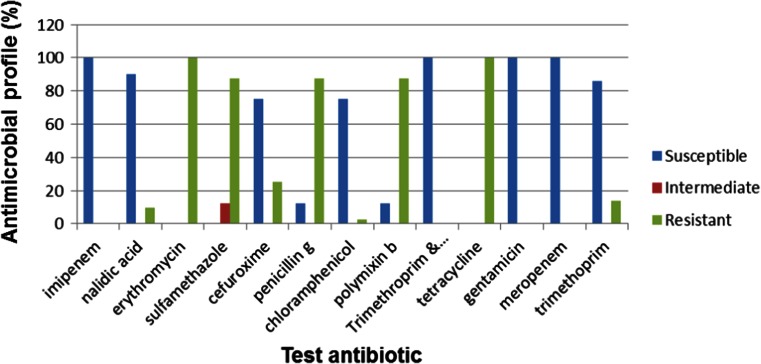
Antimicrobial profile of *V. parahaemolyticus* (*n* = 86)

Improved susceptibility to antibiotics was observed in *V. parahaemolyticus* which showed susceptibilities of ≥50 to 8 % of the test antibiotics and a resistance profile of ≥50 % to only 5 of the 13 test antibiotics (Fig. 5).

**Fig. 6:**
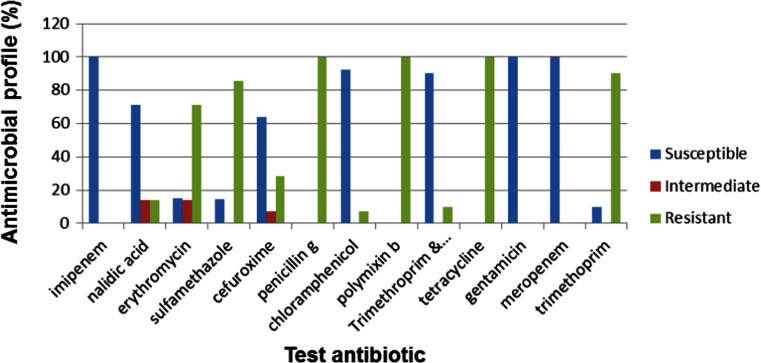
Antimicrobial profile of *V. vulnificus* (*n* = 84)

*V. vulnificus* showed a susceptibility profile of ≥50 to 7 % of the test antibiotics while showing a resistance profile of ≥50 to 6 % of the 13 antibiotics used (Fig. 6).

All three *Vibrio* species were susceptible to gentamycin, cefuroxime, meropenem and imipenem. Multiple antibiotic resistance patterns were also evident especially against such antibiotics as tetracycline, polymixin B, penicillin G, sulfamethoxazole and erythromycin against which all *Vibrio* species were resistant. Similar findings were reported by Keddy (2010a) during a cholera outbreak in South Africa in 2009, where the Enteric Diseases Research Unit (EDRU) at the National Institute for Communicable Diseases (NICD) processed 570 *V. cholerae* O1 isolates associated with the outbreak. Further laboratory characterisation of the isolates showed that they were 100 % resistant to co-trimoxazole, 48 % resistant to chloramphenicol, 100 % resistant to nalidixic acid, 3 % resistant to tetracycline and 39 % resistant to erythromycin. In another outbreak in 2008, reported from Shebagold Mine in the Ehlanzeni district of Mpumalanga Province in South Africa, 31 isolates were submitted for analysis to the EDRU, revealing that all were biotype El Tor and displayed resistance to ampicillin, amoxycillin-clavulanate, sulfamethoxazole, trimethoprim, chloramphenicol, nalidixic acid, kanamycin, streptomycin and tetracycline, which was initially the antimicrobial agent of choice in the treatment of cholera in Africa, although they were susceptible to ciprofloxacin and imipenem (Keddy 2010b; Crowther-Gibson et al. 2011).

Our findings prove that wastewater effluents are an important source of antimicrobial resistant bacteria, as reported elsewhere (James et al. 2003; Byarugaba 2004). Atieno et al. (2013) also stated that the release of pathogenic enteric micro-organisms into aquatic environments can be a source of disease when water is used for drinking, recreational activities or irrigation. It has also been noted that the prevalence of pathogenic enteric bacteria in wastewater effluents (and hence in receiving water sources) increases public health risk if the bacteria are antibiotic-resistant because of the reduced efficacy of antibiotic treatment against human diseases caused by such bacteria (Tendencia and De la Pena 2002; Wenzel and Edmond 2009). Baine et al. (1977) reported that a large water-borne outbreak involving R+ bacteria (bacteria with R factors for antibiotic resistant gene transfer) led to a large number of deaths in Mexico, partly due to the failure of the patients to respond to antibiotics of choice. The New York Times (2013) quoted Centre for Disease Control (CDC) officials as having reported that at least 2 million Americans fall ill from antimicrobial-resistant bacteria every year and that at least 23,000 die from those infections. The paper reported that one particularly lethal type of drug-resistant bacteria, known as carbapenem-resistant *Enterobacteriaceae* (CRE), has become resistant to nearly all antimicrobials on the US market, further stating that though still relatively rare, CRE causes about 600 deaths a year in the US alone. Should the proliferation of antimicrobial resistant organisms be allowed to go unchecked, society will return to a time when people died from ordinary infections. This point is further buttressed by Torrice (Undated), in an article entitled “Multidrug Resistance Gene Released by Chinese WWTP”, where he wrote:

*In recent years, increasing numbers of patients worldwide have contracted severe bacterial infections that are untreatable by most available antibiotics. Some of the gravest of these infections are caused by bacteria carrying genes that confer resistance to a broad class of antibiotics called beta-lactams, many of which are treatments of last resort. Now a research team reports that some wastewater treatment plants in China discharge one of these potent resistance genes into the environment. Environmental and public health experts worry that this discharge could promote the spread of resistance*.

There is also the possibility of antibiotic resistance genes being transmitted to autochthonous bacteria if such genes are carried by transferable and mobile genetic elements such as plasmids, thus contributing to the spread of antimicrobial resistance (Sayah et al. 2005). Development of drug resistance may be caused by the occurrence of antimicrobial agents at sub-optimal concentrations both in human bodies by continued usage and also in the wastewater matrix via leaching. The correlation between antimicrobial use and antibiotic resistance of commensal bacteria has been documented (Van den Bogaard and Stobberingh 2000). We infer, therefore, that the extent to which bacterial isolates are exposed to antibiotics before their release in the environment could be one of the reasons for the levels of antibiotic resistance shown by *Vibrio* isolates in this study. A lot has to be done, therefore, to prevent infections with multi-drug resistant organisms which find their way into the environment from WWTPs.

The public is endangered by exposure to wastewater, which happens directly or indirectly. Direct exposure routes include ingestion of contaminated water during recreational activities such as swimming, bathing and when undertaking religious ceremonies like baptisms. In the rural areas of most developing countries where the availability of piped water is limited and in most cases non-existent, the communities utilise stream/river water for drinking and other domestic uses. Indirect exposure routes include consumption of filter feeders such as molluscs which concentrate pathogenic microorganisms occurring in contaminated water (Tamburrini and Pozio 1999). In the face of climatic change and increasing water scarcity, wastewater is increasingly being considered as a new source of water for irrigation in regions where water is scarce (Blumenthal et al. 2000), exposing farmers to the risk of infection with vibrios and other waterborne pathogens. Issues of public health, as related to both water quantity and quality, are, therefore, increasingly becoming of concern.

## Conclusion

We conclude, therefore, that potentially virulent and multi-drug resistant vibrios can be found in wastewater effluents of “healthy” communities. Even though *V. cholerae* was not assessed for in this work, we have reasonable suspicion, basing on the outcome of this work, that it is also prevalent in wastewater effluents owing to the existence of sub-clinical cholera cases in communities. The prevalence of vibrios in the aquatic milieu constitutes a public health risk to people living in underdeveloped regions with no access to potable water. The use of surface water contaminated with wastewater effluents for either irrigation or recreational activities can likely pose risk of infection from waterborne pathogens. We recommend that future studies of this kind be directed at screening and characterising *V. cholerae* since it is the major pathogenic species under the genus *Vibrio* and more so since 31.8 % of the *Vibrio* isolates in this study fell outside the bracket of targeted *Vibrio* species.
